# 右肺癌系统性清扫淋巴结术后顽固性咳嗽防治方法的探讨

**DOI:** 10.3779/j.issn.1009-3419.2010.10.08

**Published:** 2010-10-20

**Authors:** 佳 黄, 清泉 罗, 阳 申屠, 晓菁 赵

**Affiliations:** 200030 上海，上海市肺部肿瘤临床医学中心，上海交通 大学附属上海市胸科医院胸外科 Department of Thoracic Surgery, Shanghai Lung Tumor Clinical Medical Center, Shanghai Chest Hospital, Shanghai Jiaotong University, Shanghai 200030, China

**Keywords:** 肺肿瘤, 系统性清扫淋巴结, 并发症, 咳嗽, Lung neoplasms, Lymphadenectomy, Complication, Cough

## Abstract

**背景与目的:**

肺癌手术系统性清扫淋巴结能一定程度上提高肺癌患者生存率，但也会造成术后并发症增多，例如乳糜胸、喉返神经损伤致声音嘶哑、顽固性咳嗽等。目前国内外尚无外科手术方法改进以减少顽固性咳嗽的发生。本研究旨在探讨前纵隔脂肪填塞残腔是否可减少顽固性咳嗽的发生。

**方法:**

2008年1月-2008年12月，依纳入标准收治右肺癌患者60例，按随机数字表随机分为使用前纵隔脂肪填塞上纵隔淋巴结清扫遗留的残腔实验组（30例）和未使用前纵隔脂肪填塞上纵隔淋巴结清扫遗留的残腔对照组（30例）。术中依据分组采取不同的方法处理上纵隔淋巴结清扫遗留残腔，记录两组患者术后第1天引流量、拔管时间、住院时间、拔管前后日间和夜间咳嗽评分等具体外科临床资料。分别随访两组患者出院后术后咳嗽情况，并指导患者填写咳嗽量化评分表和FACT-L评分表。

**结果:**

① 两组患者手术时间、术中失血量、术后第1天胸引量、拔管时间、住院天数无明显差异；②两组患者在拔管前日间、夜间咳嗽及拔管后夜间咳嗽评分无明显差异，在拔管后日间咳嗽评分有统计学差异；③两组患者术后随访咳嗽评分表评分有明显差异；④两组患者FACT-L评分中生理状况、社会/家庭状况、情感状况、功能状况、附加关注五项评分中，后三项评分两组患者有明显差异。

**结论:**

前纵隔脂肪填塞残腔能有效减少术后顽固性咳嗽的发生，提高患者生存质量。

## Abstract (en)

**Background and objective:**

The aim of this study is to analyze the impact on the cough after radical systematic mediastinal lymphadenectomy and prevention of refractory cough with medialstinal fat to fill the residual cavity after radical systematic mediastinal lymphadenectomy.

**Methods:**

Sixty patients clinically diagnosed of lung cancer were selected according to the adopt standardization, from January 2008 to December 2008. All of the patients were divided into two groups randomly: the filling-fat group and the non-fillling-fat group. The surgical information such as operation duration time bleeding volume during operation, post-operation bleeding volume were recorded. After one month, FACT-L and LCQ were completed.

**Results:**

There are no remarkably differences between the operation duration time, bleeding volume in operation and 1^st^ post-operation day's drainage volume of the two groups. There's significant difference in the scores of cough at night after taking off the chest tube, as well as in the scores of LCQ after one month and in the scores of last items of FACT-L.

**Conclusions:**

Filling the fat of the mediastinal to cover the residual cavity left by completely systematic mediastinal lymphadenectomy can reduce the refractory cough after surgery, and can also improve the quality of the life. It has no effect on the the operation duration time, bleeding volume in operation and 1^st^ post-operation day's drainage volume of the patients.

肺癌手术是否要对纵隔淋巴结进行系统的清扫，仍是目前广为研究和探讨的问题。产生不统一的主要原因在于所获得的结论多来自于对手术病例的回顾性总结。有学者^[1, 2]^前瞻性地研究系统性淋巴结清扫对非小细胞肺癌患者长期生存率的影响，结果显示，研究组生存率远远高于对照组，两组差异有统计学意义。因此，系统性清扫淋巴结是根治性肺癌手术的重要补充。

系统性清扫淋巴结能提高患者的生存率，但也造成术后并发症增多，例如乳糜胸、喉返神经损伤致声音嘶哑、顽固性咳嗽等。顽固性咳嗽对患者术后生存质量影响很大。目前国内外尚无通过改进外科手术方式来减少术后顽固性咳嗽的报道。本研究旨在探讨是否能够通过外科手术方式的改变来减少术后顽固性咳嗽的发生。我们通过使用前纵隔脂肪填塞上纵隔淋巴结清扫术后遗留之残腔，对减少术后顽固性咳嗽的发生取得满意效果，现报告如下。

## 1 材料与方法

### 1.1 临床资料

选取2008年1月-2008年12月上海市胸科医院/上海市肺部肿瘤临床医学中心胸外科收治的肺部肿物患者。纳入标准：①临床或病理诊断为非小细胞肺癌、临床分期Ⅰa期-Ⅲb期、具备手术指征者；②男女不限；③KPS≥80分；④无心脏疾病史，心功能正常，心电图无明显异常；⑤无慢性支气管炎病史，肺功能正常；⑥术前空腹血糖正常。依患者入院顺序，根据随机数字表，将患者随机分入前纵隔脂肪填塞组（简称填塞组）和非填塞组（简称对照组）。填塞组30例，其中男性25例，女性5例，年龄40岁-75岁，平均年龄（58.83± 9.45）岁；对照组30例，其中男性23例，女性7例，年龄51岁-74岁，平均年龄（61.00±5.84）岁。两组患者在年龄、肺癌分期、肺癌病理类型等方面相比较无统计学差异（*P* > 0.05）。入院后除常规检查外，均行头颅、胸部CT扫描、腹部B超检查、全身骨同位素扫描以排除远处转移。

### 1.2 手术方式

两组患者病变同侧均行系统性清扫淋巴结。两组患者的手术切口部位、长度、剖胸程序、肋骨牵引器种类、纵隔淋巴结系统清扫程度（由同一主刀医生完成）、肋骨拉拢方式、关胸过程等基本完全一致，唯独处理残腔过程有所区别。对照组对系统性清扫上纵隔淋巴结遗留之残腔不做任何处理（图 1A）。填塞组用电刀游离一部分带蒂纵隔脂肪，将之填塞入系统性清扫上纵隔淋巴结时遗留的残腔，并用福爱乐医用胶[福爱乐医用胶是一种人工合成无色透明的以α-氰基丙烯酸正辛酯（NOCA）、α-氰基丙烯酸正丁酯（NBCA）组成的生物医学工程材料，二种单体的纯度均在99%以上]固定游离之纵隔脂肪（图 1B）。

**1:**
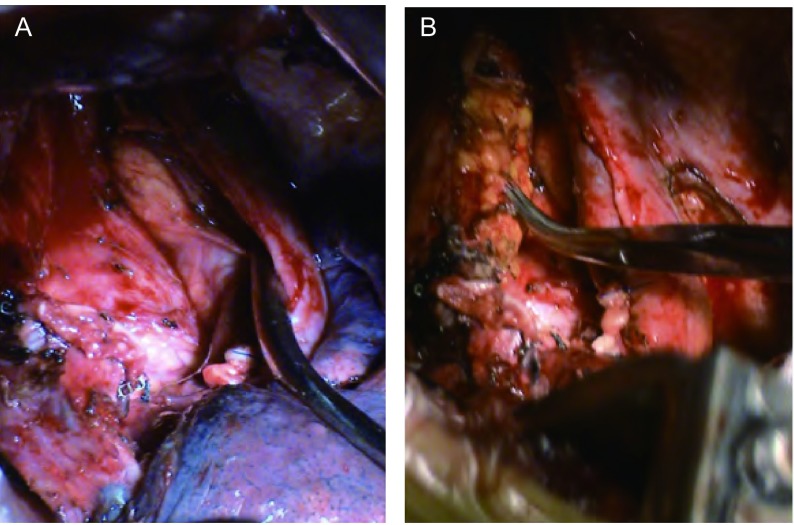
处理残腔的具体手术方式。A：不做任何处理；B：用脂肪填塞。 Different surgical handles on the residual cavity left. A: no special treatment; B: using the medialstinal fat to cover the residual cavity.

### 1.3 统计学处理

采用SPSS 12.0进行统计分析，组间计量资料比较采用独立样本*t*检验；频数资料分布比较采用卡方检验，*P* < 0.05为差异有统计学意义。

## 2 结果

### 2.1 两组患者手术相关因素的比较

本组资料显示（表 1），两组患者在术后第1天引流量、手术时间、术中出血量、住院时间、拔管时间等方面无统计学差异。可能与前纵隔脂肪仅覆盖于清扫第1组-第4组淋巴结遗留之残腔，该残腔面积较小，胸水渗出量也较少，虽然覆盖了纵隔脂肪，但其减少的渗出量有限，因此对术后引流量、术中出血量等指标基本不造成影响。说明该手术方法的改进不会增加术中出血量，亦不会延长手术时间和住院时间。

**1:** 两组患者手术相关因素的比较 Comparison on surgical results between 2 groups of patients

Groups	Operation time (min)	Operative bleeding volume (mL)	Drainage first day (mL)	Extubation time (day)	Postoperative hospitalization (day)
Non-filling-fat group	126.85±33.40	312.96±115.90	368.15±170.52	6.1±6.13	10.3±3.1
Filling-fat group	134.44±25.24	268.52±84.52	416.30±137.70	5.1±2.48	9.8±1.5
*P*	0.350	0.530	0.259	0.385	0.081

### 2.2 两组患者术后并发症情况

#### 2.2.1 拔管前后日间与夜间咳嗽评分比较

使用咳嗽评分表（分值越低，咳嗽越少），对两组患者术后咳嗽情况进行评分。结果显示，在拔除胸管前，填塞组与对照组两组患者日间及夜间咳嗽情况差异无统计学意义（*P* > 0.05）（表 2）；而在拔除胸管后，两组患者日间咳嗽情况差异无统计学意义（*P* > 0.05），但填塞组夜间咳嗽情况明显好于对照组，差异具统计学意义（*P*=0.004）（表 3）。

**2:** 两组患者拔管前日间与夜间咳嗽评分比较 Comparison of cough score between day and night before taking off the chest tube

Groups	Cough scores during the day				*P*	Cough scores at night				*P*
	0’	1’	2’	3’		0’	1’	2’	3’	
Non-filling-fat group	25	5	0	0	0.563	18	12	0	0	0.093
Filling-fat group	26	4	0	0		16	12	2	0	

**3:** 两组患者拔管后日间与夜间咳嗽评分比较 Comparison of cough score between day and night after taking off the chest tube

Groups	Cough scores during the day				*P*	Cough scores at night				*P*
	0’	1’	2’	3’		0’	1’	2’	3’	
Non-filling-fat group	16	10	3	1	0.004	16	14	0	0	0.365
Filling-fat group	10	11	6	3		17	13	0	0	

#### 2.2.2 患者咳嗽评分随访情况

术后1个月随访两组患者，指导患者完成术后咳嗽评分表，两组患者咳嗽平均得分见表 4，填塞有助于改善咳嗽症状和患者生存质量（*P* < 0.05）。

**4:** 术后1个月随访两组患者咳嗽评分情况 The cough scores of patients in two groups one month after the operation

Item	Non-filling-fat group	Filling-fat group	*P*
Sick to stomach	6.47±0.57	6.43±0.68	0.592
Have you been bothered when you cough	3.33±0.99	4.26±1.01	0.025
Have you been tired because of cough	3.53±0.69	4.16±0.98	0.304
Have you felt in control of your cough	4.01±0.62	4.11±0.71	0.059
Have you felt embarrassed by cough	3.13±0.76	4.06±1.11	0.415
Cough has made me feel anxious	3.07±0.59	3.96±0.91	0.001
Cough has interfered with daily tasks	3.43±0.99	4.36±0.81	0.323
Cough interfered with the overall enjoyment of my life	3.13±0.79	4.01±0.76	0.005
Exposure to paints or fumes has made me cough	5.01±0.66	4.89±0.77	0.117
Has your cough disturbed your sleep	2.92±0.69	3.93±0.81	0.003
How many times a day have you had coughing bouts	3.03±0.82	4.16±0.87	0.037
Cough has made me feel frustrated	3.43±0.62	4.36±0.88	0.042
Cough has made me feel fed up	4.04±0.71	3.99±0.68	0.945
Have you suffered from a hoarse voice as a result of your cough	4.43±0.83	4.38±0.74	0.673
Have you had a lot of energy	3.01±0.62	3.11±0.71	0.068
Cough has interrupted conversation or telephone calls	3.25±0.85	4.16±0.78	0.034
Cough has annoyed my partner, family or friends	4.21±0.77	4.44±0.86	0.070
Scores	63.43±10.11	72.77±8.75	0.036

#### 2.2.3 两组患者生存质量情况

术后1个月随访两组患者，指导患者完成生存质量表，两组患者生存质量平均得分见表 5，填塞组在情感状况、功能状况及附加关注等方面要优于对照组，两组差异具统计学意义（*P* < 0.05）（表 5）。

**5:** 术后1个月两组患者随访生存质量情况 The quality of life of patients in two groups one month after the operation

Groups	Physical condition	Social/family situation	Emotional condition	Functional status	Additional concerns
Non-filling-fat group	18.7±5.4	23.6±3.8	18.3±2.4	19.2±2.0	24.4±3.2
Filling-fat group	18.3±5.7	23.9±4.4	16.6±2.1	17.5±2.4	22.0±2.8
*P*	0.381	0.485	0.006	0.006	0.003

## 3 讨论

在肺癌手术中，“是否行系统性肺门纵隔淋巴结清扫术”是广受争议的话题。目前研究^[3, 4]^认为，越是早期的肺癌病例，越是需要进行纵隔淋巴结的清扫术。系统性清扫淋巴结能提高患者的生存率，但也造成术后并发症增多，例如乳糜胸、喉返神经损伤致声音嘶哑、顽固性咳嗽等。顽固性咳嗽对患者的生存质量影响很大，是否可以通过改进手术方式改善咳嗽情况值得研究。

目前认为肺癌术后顽固性咳嗽发生主要是因为^[5, 6]^，肺癌患者在清扫淋巴结的过程当中，特别是在清扫隆突下淋巴结和上纵隔淋巴结的时候，摘除淋巴结之后留下空腔。而快速适应性肺部牵张感受器就位于隆突下和主支气管周围。由于空腔形成，使得这些感受器暴露在外，人体的活动造成的机械性牵拉和术后胸水都能刺激到这些感受器，然后通过有髓鞘的Aδ纤维传导，能将这些机械性和化学性刺激经迷走神经传入脑干，然后由迷走神经内的运动纤维传出，形成咳嗽反射。因此，使用前纵隔脂肪填塞清扫上纵隔淋巴结遗留之残腔的方法尝试观察患者术后咳嗽情况是有解剖学和生理学依据的。

本研究中，两组患者的手术切口部位、长度、剖胸程序、肋骨牵引器种类、纵隔淋巴结系统清扫程度（由同一主刀医生完成）、肋骨拉拢方式、关胸过程等完全一致，唯独处理残腔过程有所区别。填塞组用纵隔脂肪填塞清扫上纵隔淋巴结遗留之残腔。对照组则对残腔不做任何处理。从操作角度讲，对照组相对快捷，但填塞组手术过程中整个操作过程平均耗时3 min左右。两组手术时间、术中失血量、术后第1天引流量、拔管时间、住院时间属于操作固有的衡量指标，应能较客观地反映手术本身对患者的创伤程度，数据显示两组并无显著性差异，换言之，用纵隔脂肪填塞清扫上纵隔淋巴结遗留之残腔不会增加术中出血量，亦不会延长手术时间和住院时间，未增加手术患者的创伤。因为所使用纵隔脂肪为带蒂组织，因此丰富的血供保证脂肪组织日后不会坏死和液化。术后胸部CT显示上纵隔软组织影，部分影像科医生会误将之视为淋巴结肿大，需做好沟通，以指导内科医生制定下一步治疗方案（是否联合放疗）。在试验后期，我们也曾使用明胶海绵填塞残腔，并缝合两侧胸膜，关闭残腔，也取得不错效果。笔者认为后者方法更可取，毕竟游离纵隔脂肪也增加了创伤，尽管两组患者在术后创伤程度方面并无显著性差异。

此手术方式在改进对咳嗽的改善方面，拔管前两组患者日间与夜间咳嗽评分无明显差异，可能是因为拔管前患者有胸管置入胸腔，害怕活动后移动胸管引起疼痛，故较制动，而且胸水无法积聚于胸腔内，一有胸水即会从胸管内溢出，故机械性牵拉和胸水刺激较少，咳嗽就不明显，两组患者之间咳嗽就无明显差异。而拔管后两组患者日间咳嗽评分存在明显差异，原因可能为，拔除胸管后，瞩患者下床活动，患者活动增多，胸水产生，积聚于胸腔内。人体的活动刺激咳嗽感受器引起咳嗽。因此患者拔管后咳嗽增多。而拔管后两组患者夜间咳嗽评分无明显差异，可能是因为夜间活动较少，刺激人体咳嗽感受器的机率就大大减少所致。填塞组由于用前纵隔脂肪填塞清扫上纵隔淋巴结遗留之残腔，使胸水和机械性牵拉无法刺激感受器，故减少咳嗽的发生。

术后咳嗽评分表来源于Leicester咳嗽问卷（Leicester Cough Questionnaire, LCQ）^[7]^。LCQ是一个简单、可自测、特异性和真实性好的咳嗽健康相关生活质量问卷，由19个项目组成， < 5 min就可完成该问卷。早期使用LCQ的经验表明，患者对问卷的回答和数据的完整性均很好。LCQ的最小重要性差异为1.3，这代表了患者觉得有意义的最小健康状态变化。LCQ已被成功运用于临床试验和其它研究中，初步经验表明，LCQ可成功地用于评估肺癌术后患者的咳嗽状况，故现将LCQ中19个项目调整为17个，用来评价患者术后咳嗽情况^[7]^。术后1个月随访术后咳嗽评分表，两组在被咳嗽、咳痰而困扰、因为咳嗽而感到疲倦、咳嗽经常使您感到窘迫、咳嗽使您感到焦虑、咳嗽使您感觉生活不便、咳嗽有影响到您的睡眠等选项中存在明显差异（*P* < 0.05）。该评分体现了对照组患者在生理、心理、社会三方面都受到了术后咳嗽的影响，而填塞组相对于对照组来说有一定优势，但是不可否认的是，还是影响了患者的正常生活，只是影响减少了。

目前认为^[6, 8]^肺癌患者生存质量的内容应包括：①生物学：如疾病症状、治疗副作用和机体功能状况；②心理学：指确诊为肿瘤后对病人心理的影响及病人对此疾病所做出的反应；③社会学：如社会关系、工作能力、经济支持及医疗情况。FACT是由美国芝加哥Rush_Presbyterian_St. Luke医学中心的Cella等研制出的癌症治疗功能评价系统（Functional Assessment of Cancer Therapy, FACT）^[9]^。其中，FACT_L（V4.0）由FACT_G的27个条目和肺癌附加关注的9个条目构成，专门用于肺癌患者的生存质量测定。全表采用自评的方式填写。此表在临床的使用也相当广泛，用于临床Ⅱ期、Ⅲ期试验，可全面评估肺癌患者的生存质量，已通过了效度、信度及反应度的检验^[10, 11]^。故将该表用于术后1个月随访生存质量。结果显示，在生理状况、社会/家庭状况两项无明显差异（*P* > 0.05），情感状况、功能状况、附加关注后三项评分中都有统计学差异（*P* < 0.05），对照组因为顽固性咳嗽情绪更加紧张，而且在疾病的抗争过程中越来越失望，并担心疾病是否加重，晚上睡眠质量较差，基本无法享受生活，抱怨一直在咳嗽。而填塞组患者在这几项评分中要明显高于对照组，在术后生存质量方面要优于对照组。目前的医学模式已从以前的生物医学模式转化为生物-心理-社会医学模式，对于患者，不仅要延长患者的生存期，还要提高患者的生存质量。从两组患者的术后1个月反馈的信息看来，填塞组较对照组提高了患者的生存质量。

综上所述，本研究证明了用前纵隔脂肪填塞清扫纵隔淋巴结遗留之残腔不增加患者创伤，能有效减少术后顽固性咳嗽的发生，显著提高了肺癌患者术后的生存质量，是肺癌手术可取的改进方式。但因本研究样本量较小，尚有待进一步深入研究。
